# The long-term impact of a comprehensive scholarly concentration program in biomedical ethics and medical humanities

**DOI:** 10.1186/s12909-018-1311-2

**Published:** 2018-08-28

**Authors:** Emily Yang Liu, Jason Neil Batten, Sylvia Bereknyei Merrell, Audrey Shafer

**Affiliations:** 1 Hospital of the University of Pennsylvania, 3400 Spruce Street, Philadelphia, PA 19104 USA; 2 Stanford School of Medicine, 291 Campus Drive, Palo Alto, CA 94305 USA; 3 Department of Surgery, 300 Pasteur Drive, Room H3552, Stanford, CA 94305 USA; 4 VA Palo Alto Healthcare System, 3801 Miranda Avenue, Anesthesia 112A, Palo Alto, CA 94304 USA

**Keywords:** Medical education, Healthcare education, Professional development, Bioethics, Medical humanities, Health humanities

## Abstract

**Background:**

There is a strong and growing interest in biomedical ethics and medical humanities (BEMH) within medical education for facilitating key components of medical professionalism and ethics, clinical communication and observational skills, and self-care and reflective practices. Consequently, United States (US) medical institutions have begun to incorporate BEMH through formal Scholarly Concentrations (SCs). This is the first study to examine the impact of a US BEMH SC, from student experience in medical school to post-graduate development, as perceived by graduate physicians.

**Methods:**

Graduated students who participated in the BEMH SC or did extensive BEMH research prior to the BEMH SC’s establishment (*n* = 57) were sampled for maximum variation across graduating years. In telephone surveys and interviews, participants discussed the perceived impact of the BEMH SC on (a.) student experience during medical school and (b.) post-graduate development. Interviews were audiotaped, transcribed, and de-identified. The authors iteratively generated a codebook; two raters coded independently, adjudicated codes, and completed inter-rater reliability (IRR) tests. The authors subsequently conducted a team-based thematic analysis, identifying emergent themes.

**Results:**

Nineteen BEMH graduates were interviewed. Results were analyzed according to (a.) student experience and (b.) post-graduate development. Overall, respondents perceived impacts in reinforcing knowledge and skills in clinical ethics; solidifying self-care and reflective practices; refining a sense of professional identity and integrity for ethically challenging situations; and promoting student skills, productivity, and later careers involving BEMH.

**Conclusion:**

A comprehensive US BEMH SC achieved the purported aims of BEMH in medical education, with graduate physicians perceiving persisting effects into clinical practice. Furthermore, the structure and format of a SC may offer additional advantages in promoting student scholarly skill and productivity, career development, and professional identity formation—core competencies identified across clinical training and ethics programs. Our findings indicate that a BEMH SC is effective in achieving a range of desired immediate and post-graduate effects and represent a particularly promising venue for BEMH in medical education. We believe these findings to be of critical significance to medical educators and administrators when considering how best to incorporate BEMH into SCs and medical curricula.

**Electronic supplementary material:**

The online version of this article (10.1186/s12909-018-1311-2) contains supplementary material, which is available to authorized users.

## Background

Since the term “medical humanities” was introduced in the United States (US) in the 1960s, there has been strong and growing interest in biomedical ethics and medical humanities (BEMH) within medical education [1–6]. Concurrently, controversy exists regarding the role and scope of medical humanities (e.g., additive versus integrative to medicine), the relationship between biomedical ethics and medical humanities (e.g., are they a singular entity, separate entities, natural bedfellows?), and how BEMH is best implemented (e.g., electives vs. required coursework) amongst other debates [2–5, 7–9]. Regardless, there is strong consensus around its fundamental aims: encouraging key components of medical professionalism (e.g., empathy, altruism) and ethics, honing clinical communication and observational skills (focusing on emotional and non-verbal cues, the cultural context of care, and elucidating patient values), and developing self-care and reflective practices—foundational skills for physicians [1–9]. Consequently, numerous US academic medical institutions have begun to incorporate BEMH in various forms within their curricula [10–13].

Simultaneously, there is a trend toward Scholarly Concentrations (SCs) within medical curricula in the US. An SC (alternatively “track” or “area”) is a series of elective or required courses, or other curricular experiences, beyond the core medical curriculum for students to study a specific subject in-depth [14]. BEMH has increasingly assumed the form of SCs in medical education in the US, with 12 schools offering bioethics and/or medical humanities as SCs [15].

Given the increasing thrust toward BEMH SCs in the US, insight into the capacity of BEMH SCs to achieve the purported aims of BEMH in medical education is particularly relevant for medical educators and administrators. However, no study to date has evaluated the impact of a comprehensive BEMH SC, from student experience in medical school to post-graduate development, as perceived by graduate physicians. Here, we present findings from an exploratory qualitative study on the perceived impact of Stanford’s BEMH SC in terms of (a.) student experience during medical school, and (b.) post-graduate development.

### The program

Stanford’s SC program was formally established in 2003 [16]. Completion of a SC is required for all MD candidates, except for those in the MD-PhD track. Students select one of eight SCs; some may additionally elect to focus in one of seven application areas as an adjuvant to their SC [17]. An overarching description of Stanford’s SC program is provided in Laskowitz et al. [16]

The BEMH SC is one of the original SCs; it consists of curricular, research, scholarly, and extracurricular experiences spanning pre-clinical and clinical years (Table 1). Approximately 10% of MD students select the BEMH SC each year, with an additional 5% of students completing funded projects through BEMH mentorship. Unique to the BEMH SC, the required scholarly project can be in the form of traditional research or substantive creative work (e.g., novel, short film); the two receive equivalent treatment in terms of evaluation, academic credit, faculty mentorship, and financial support through Stanford’s Medical Scholars Research Program [18]. Funding through Medical Scholars is per project and allocated on an academic quarter system by the School of Medicine; students are eligible for a maximum of 5 quarters of funding at 100% effort or at a prorated scale for part-time research quarters [18]. Development of Medical Scholars projects in medical humanities occurred before the establishment of the SC program and provided time to hone criteria and build mentorship networks [19, 20].

**Table 1:** The Stanford Biomedical Ethics and Medical Humanities Scholarly Concentration (BEMH SC)

	Year 1	Year 2	Year 3	Year 4	Research Year (optional)
BEMH coursework • Introductory humanities and ethics courses: ethics courses involve shadowing ethics consults and attending ethics committee meetings • Additional electives from across the university • Total of 12 course units	Most students complete required and elective courses within the first two years of medical school (preclinical years)		Students may complete additional courses during clinical years.		Students may elect to take an additional research year at any point during medical school. In addition to dedicated time for work on continuing or new projects, they may participate in coursework and extracurricular activities during their research year.
Research • Scholarly project required for graduation: includes required oral presentation (can be conference presentation) and written report (can be publication) • Competitive grant support and advising through Medical Scholars Research program • Can be in the form of traditional research or creative projects	Most students complete requirements for a scholarly project within the first two years of medical school (preclinical years).		Students may use clinical years to continue projects from the first two years of medical school or pursue additional projects.		
Extracurricular activities • Consists of a variety of activities: writing workshops/journals, student art symposium, lecture series (e.g., on end-of-life care), etc.	Students can and do participate in extracurricular activities throughout all four years of medical school.				

Components of the BEMH SC, including coursework, research, and extracurricular activities, organized by program year

The Stanford BEMH SC is a comprehensive program and one of the earliest of its kind, allowing us to uniquely examine the persisting impact of the BEMH SC across clinical training stages.

## Methods

In this study, we qualitatively explored how a dedicated and comprehensive curriculum in BEMH impacted (a.) student experience during medical school and (b.) post-graduate development, as reported by past graduates. All graduated medical students who completed the BEMH SC or who did extensive research prior to the establishment of the SC between 2007 and 2014 at the study institution were eligible to participate. The study was conducted over telephone and consisted of a structured demographic survey followed by an in-depth, semi-structured interview. We purposefully sampled for maximum variation across eligible graduates (*n* = 57) [21].

One author (EYL) conducted all telephone encounters; after each encounter, the interviewer wrote memos about the interview to inform the research process. Prior to the start of data collection, we iteratively refined the interview questions and subsequently piloted the interview guide for understandability— performing minor adjustments in the language of questions as needed. We conducted the surveys/interviews until thematic saturation was reached, as defined by participants’ repetitive comments and the lack of emergent themes, essentially indicating no new information [22]. The structured survey consisted of questions on participant characteristics (e.g., entering and graduating years, choice of specialty) and validated demographic questions from the US census [23]. The semi-structured interview guide probed relevant perceived impacts for BEMH as well as SC programs derived from the literature; however, interviews were intentionally semi-structured to facilitate an open-ended examination of participants’ experiences and perspectives beyond identified outcomes [5, 14, 24]. Please see Additional files 1 and 2 for the telephone survey and interview guide respectively.

Each audiotaped encounter (surveys, interviews) was transcribed verbatim by an outside transcriber and validated and de-identified by our research team. The primary author (EYL) generated an initial codebook through an inductive approach that was iteratively refined by all authors [25]. Two authors (EYL, JNB) underwent three staggered inter-rater reliability tests of 50% of the transcripts to ensure consistent application of the finalized codebook between coders throughout the study and as a method of triangulating the accuracy of the findings [26]. A minimum standard of Cohen’s pooled Kappa of 0.7 was pre-determined to be the acceptable inter-rater reliability between two coders [27–29]. Two authors (EYL, JNB) independently applied final codes to all transcripts facilitated by Dedoose Qualitative Analysis Software Version 5.0.11 (SocioCultural Research Consultants, LLC; Los Angeles, CA) Afterward, we conducted a team-based thematic analysis, identifying and refining emergent themes through discussion [30].

This exempt study protocol was approved by Stanford’s Institutional Review Board.

## Results

Nineteen BEMH graduates were interviewed prior to data saturation, as evidenced by the absence of new insights or concepts from additional interviews through coding and reviewing thematic ideation via memos. Specifically, no new codes emerged in the final 5 interviews, suggesting that the overarching concepts were consistent across respondents’ experiences. Respondents spanned all graduating years with representation of males and females. Multiple specialties were captured, from primary care-oriented fields (e.g., family medicine, internal medicine, pediatrics) to specialized fields (e.g., plastic surgery, dermatology). Participant characteristics are provided in Table 2. The three inter-rater reliability tests of the codebook resulted in κ 0.88, 0.87, and 0.91, and were deemed to be sufficient [27].

**Table 2:** Participant characteristics

Years since graduation (at time of study)	
Range	1–8
Median	7
Year of graduation	
2007	21% (*n* = 4)
2008	26% (*n* = 5)
2009	5% (*n* = 1)
2010	11% (*n* = 2)
2011	5% (*n* = 1)
2012	5% (*n* = 1)
2013	16% (*n* = 3)
2014	11% (*n* = 2)
Sex (% male)	32% (*n* = 6)
Specialties	
Psychiatry	21% (*n* = 4)
Pediatrics	21% (*n* = 4)
Family medicine	16% (*n* = 3)
Emergency medicine	11% (*n* = 2)
Internal medicine	5% (*n* = 1)
Dermatology	5% (*n* = 1)
Plastic surgery	5% (*n* = 1)
Anesthesiology	5% (*n* = 1)
Radiology	5% (*n* = 1)
Pathology	5% (*n* = 1)

Years since graduation, year of graduation, sex, and specialty of BEMH SC study participants (total = 19)

Perceived impacts spanned medical school and post-graduate years; thematic analysis focused on (a.) student experience during medical school, and (b.) post-graduate development.

We found some variability in participant responses regarding their experiences in the BEMH SC, likely indicative of program flexibility for the pursuit of individual interests as well as the program’s evolution over time. Moreover, some individuals felt the BEMH SC had minimal impact. Others had difficulty discerning the impact of the BEMH SC from preexisting qualities (e.g., an interest in the arts/creative writing, undergraduate experience) or other aspects of training (e.g., palliative care fellowship). In our results, we present findings repeatedly identified across multiple respondents.

### Student experience

Overall, participants described the BEMH SC as a structured, longitudinal scholarly experience with dedicated mentorship, financial, and time support that encouraged reflective practices and the pursuit of personal interests. They discussed their experience of (1.) core curricular and extracurricular activities within the BEMH SC and (2.) individual scholarly projects. Illustrative quotes are provided in Tables 3 and 4.

**Table 3:** Student Experience—Core Curricular and Extracurricular Activities

*The core coursework provided theoretical and practical exposure to BEMH* I remember sitting in on ethics committee meetings at the hospital, being able to see how the things that we were learning about bioethics were being applied in a real-world situation. (I-4) The creative writing for medical students course...was a great way to force myself to really write…to focus more formally on creating a finished or semi-finished product that I could share with others and get feedback.... My writing got very, very, very strong…. Those skills in writing I feel like carry on into my clinical work quite a bit. (I-6)	
*The core coursework provided a valuable experience not otherwise available in the medical school curriculum* The coursework was very interesting…. It was both medical humanities and ethics. I liked that it supplemented our regular education because otherwise we…didn’t really get any in our curriculum. (I-14) Communication skills through the BEMH concentration are crucial.... It’s the students who do not take any classes who could use it the most.... They struggle [in] the wards, they struggle with sort of complex communication, with having difficult conversations. (I-1)	
*The program’s flexibility allowed for exploration of individual interests through elective courses* BEMH was really flexible in letting me take the classes that I actually was interested in. (I-12) Many of my colleagues combined other interests say in women’s health or palliative care.… [BEMH] can lend itself to any sort of medical specialty, so I think flexibility is one of the things that stood out about the program. (I-1)	
*The curriculum provided opportunities to engage in reflective and self-care practices* Once a week…we would do some free writing…we would listen to what people had to read and…reflect on our medical training experiences in a very non-threatening, supportive environment. (I-10) A very meaningful part or topic that came up [in the SC]…was…work-life balance and risks of burning out or addiction as a physician…taking care of oneself at the same time as taking care of other people. That’s a very real issue for anyone going forward in medicine. (I-5)	
*The SC provided a safe and nurturing community for students* Things that were…more special about [the BEMH SC]: the community, the sense of home, and the space to…think…more creatively in these…arenas…everybody, all the faculty in BEMH felt like…both genuine people and committed people…so that felt like a safe place…where…those part of me could come out. (I-2) You get plenty of your intellectual feeding in medical school…and plenty of discipline feeding…[but] you don’t get sometimes that soul nourishment that keeps the whole machine ticking. I had that [with the BEMH faculty and community]. (I-18)	

Illustrative quotes from participants on the perceived impact of the BEMH SC on their student experience, related to core curricular and extracurricular activities

**Table 4:** Student Experience—Scholarly Projects

*Students were empowered from the financial support and protected time they had to pursue personal interests* This was in the Medical Scholars Program before they had the SC, but the support that I enjoyed...was unparalleled…financially which was important for me, but also access to other parts of the university and other faculty...and the general acknowledgement of the importance of arts and humanities within medicine. (I-11)	
*Projects provided opportunities for reflective and self-care practices* [My scholarly project] required me to think … critically about myself and think about the forces and the influences on my life in a very structured format, but then also thinking about the impact I might have on the people around me…. It almost left me in a very centered place that I feel in many ways increased my resiliency to the challenges of the clinical years of medical school. (I-6) I remember on one particular rotation...I actually had six of the patients that I was following pass away…. I realized that I didn’t really know what it felt like to be in the patient’s shoes and so wanted to explore that component…that’s how I came to the idea of creating a play set to music that we could basically perform…and invite people to come and experience. (I-4)	
*Students received encouragement and personalized guidance from mentors on projects* [My mentors] were all really supportive, and it’s just pretty fun to do like, “Yeah, I kind of want to make this comic book,” and then people would take you seriously and support you. I think they’re really great mentors, and we’re all lucky to have them. (I-3) I feel like it’s hard…for me to emphasize…how wonderful [my mentor] was...I feel like she really made an incredible effort to try to understand what I was trying to say and then help me to see ways where I could try to make my writing stronger.... She did an amazing job of …connecting us with each other and with other faculty mentors who could help us. (I-6)	
*Students achieved scholarly products (e.g., manuscripts, speaking engagements) through their projects* My MedScholars project was a novel, it was…a medical thriller, a medical mystery novel that was then bought by [a publishing house]…I got a two book deal, so my MedScholars project was a pretty significant one…. It was a novel. (I-11)	
*Students developed scholarly skills through their projects* I had to both propose and apply for the funding and then the body of the research itself, I had to become…familiar with methods of photographic research and narrative medicine and audio, like taking oral histories, so doing that and then actually creating a project. (I-18) Even just writing a proposal up of my own idea...how do I convince this group that they want to sponsor me to do this…figuring out what you want to do and seeking out a mentor, all of those...skills apply moving forward for any project. (I-17)	

Illustrative quotes from participants on the perceived impact of the BEMH SC on student experience, related to scholarly projects

#### 1. Core curricular and extracurricular activities

Speaking to the rigor of the program, respondents commented that it manifested in the breadth of exposure and regular opportunities for in-depth engagement with BEMH. Specific activities included classroom ethics case discussions, hospital ethic consults and committee meetings, and creative writing classes and workshops. Multiple participants felt these to be positive learning experiences that enabled them to appreciate the relevance of BEMH to clinical practice as students.

Moreover, respondents felt that the SC provided learning experiences otherwise unavailable through the general curriculum, which they described as “science-heavy” (I-4). Many appreciated the opportunity to explore “the softer…humanistic side of medicine” (I-4).

One individual wished this exposure was accessible to students outside the BEMH SC for its positive impacts on complex communication skills in clinical years. Moreover, many appreciated the flexibility of the BEMH SC, which they felt was above what was available through Stanford’s general medical curriculum and other SCs. Respondents were able to take a range of courses (e.g., health economics, photography) and combine their coursework in the BEMH SC with individual interests (e.g., women’s health, palliative care).

Respondents found their experiences in BEMH SC curricular and extracurricular activities enabled them to develop and cultivate self-care and reflective practices. For many, these consisted of particular activities—e.g., writing—that they participated in through classes, but also scholarly projects. For others, it had more to do with specific humanistic perspectives, focusing on work-life balance and finding meaning through work, with one respondent explaining, “things that are humanities-based…naturally there’s more of that kind of focus” (I-3).

Lastly, respondents appreciated the safe and nurturing community the BEMH SC provided, where they could engage with like-minded individuals. Many developed close personal relationships with faculty and perceived the BEMH SC not only as a community but a “home…to think more creatively…a safe place” (I-2). A few attributed life-long relationships with other classmates to their shared time in the BEMH SC.

#### 2. Scholarly projects

With regards to financial and time support through the BEMH SC, respondents commented it enabled them to pursue personal interests they otherwise might not have. One individual explained, “I wanted to try my hand at…a true writing project. I’ve always enjoyed creative writing but I’ve never, you know, received funding or anything…I don’t think I would have received funding to write … if I were just out in the community, so that was…something that I really enjoyed” (I-14). Another was able to complete two novels during medical school in the protected time for scholarly projects as well as weekends, vacations, and holidays. Multiple respondents commented that the degree of support given to BEMH SC projects fostered a deeper appreciation of the relevance and significance of BEMH to medicine. As one individual described, it was a powerful acknowledgement by the School of Medicine “that not everybody sort of fits into…traditional molds, and that it was…just as valued to…pursue work within the…softer science fields” (I-17).

Respondents reported scholarly projects provided further opportunities to engage in self-care and reflective practices. For example, one project by an African-American BEMH SC graduate was a narrative account, reflecting on personal and professional experiences of race and racism in medicine.

For these self-directed projects, participants greatly valued the support and personalized guidance provided by mentors, with one respondent applauding BEMH SC mentors for their “strong individualized interest in students, not only in their…research but in their careers…as well as them as individual people” (I-1). Respondents appreciated that project advisors regarded their work “seriously”, no matter how non-traditional. Many remarked that mentorship was a great strength of the BEMH SC.

Lastly, respondents were able to develop tangible skills and products through their scholarly projects. Many presented or published work at academic conferences and in peer-reviewed journals as medical students. One individual signed a multi-book contract for a medical thriller series whereas another was invited to speak at a presidential committee. Additionally, individuals felt they developed critical scholarly skills in conceptualizing, designing, and implementing projects—“how to frame study questions” and “how to do data analysis on more qualitative research” (I-15)—as well as specific research methodologies, such as narrative medicine. Some were connected to valuable resources and collaborators. A few felt that the grant application process was a useful learning experience; other skills cited by respondents included manuscript preparation and increased familiarity with the publishing process.

### Post-graduate development

While the BEMH SC was clearly perceived to impact student experience during medical school, respondents also felt that it had long-term implications on post-graduate development in terms of (1.) clinical practice, and (2.) career decisions and lifestyles. Representative quotes are provided in Table 5.

**Table 5:** Post-graduate development

*Incorporating BEMH into clinical practice* Graduates have increased familiarity and comfort with bioethics and clinical ethics The big ethical issues come up every day in clinical practice and research…. The training that we got, especially…hands-on doing consults with…the ethics committee was very helpful. (I-8) [Though] plastic surgery is based on a set of rules, how you use those rules is completely dependent on how you...think as an individual. The sort of things that you learn in the SC are …how you are able to think about various things outside of the hard confines of…hard science…[to consider] whatever else they’ve been through in their lives or how compliant they are, how old they are, what their living situation is. (I-7)	
Graduates incorporate narrative in appreciating patients as individuals. I think being able to think about each person…[as] having a story that’s unique...is helpful in talking to the patient, gathering information and really understanding who they are…[and] also in communicating to colleagues because humans are such narrative creatures. (I-10) In the same way…as being able to communicate with someone, observing…. I’m thinking of two things…it’s truly effective in helping with the physical exam in that context of observation, but also observing people…their thoughts, interacting with them, observing their emotions. (I-5)	
*Career decisions and lifestyle* Graduates value and practice self-care, wellness, and work-life balance I really strongly believe in work-life balance, and it’s something hard to achieve in a medical career, but I think I’m doing okay with that, and I’m sure it has something to do with my experience in the concentration.… I think in order to be a good doctor you have to take care of yourself as well, particularly because we see so much sad stuff all the time, and sometimes being able to reflect on it by writing or by discussing like we did in our classes can be really therapeutic and help make you a better doctor. (I-14) [The BEMH SC has] affected my planning for the future…. I don’t want to devote my time solely to working 24/7, I would like to balance my life with some kind of more humanistic literary/writing type of work, which I think offsets the potential of burning out. (I-12)	
Graduates’ time in BEMH influenced their choice of specialty Knowing I…was very interested in writing and having time to write and join writing groups… made me want to find a specialty that…would give me the time and space…to be able to step back from my clinical work and think more broadly about myself in medicine and some of the challenges from a more macro level…. I feel like that certainly affected the field that I chose…trying to find the right balance. (I-6)	
Graduates had positive role models for careers in or incorporating BEMH A lot of the faculty within the concentration are not…super traditional…they have…these outside interests that are…quirky for lack of a better word…. Seeing that and interacting with people like that made me appreciate that you could have…an alternative perspective on medicine but still be a very rigorous, very good clinician-scientist. (I-9)	
Some graduates pursued careers in or incorporating BEMH There’s the effect of having a couple of people that are trained and confident in this aspect of medicine…it’s a little bit infectious, it changes the culture of the department and a residency…in a very healthy way…. If I had been in a medical school [without] a medical humanities department, it wouldn’t have occurred to me that this was something I could do, that it was needed and that there’s a place for it and that it can be rigorous. (I-18) I’m building a communication curriculum for our pediatric residents…arising out of a needs assessment…. It’s something that from the beginning of my medical career was extremely important, and sadly, I don’t think is prioritized as much as other knowledge. (I-17)	

Illustrative quotes from participants on the perceived impact of the BEMH SC on post-graduate development

#### 1. Incorporating BEMH into clinical practice

Regarding increased familiarity and comfort in bioethics and clinical ethics, participants described this as a “formalized way of thinking” (I-13), a “framework” (I-1, I-18), and “a sense of comfort talking about issues of autonomy, futility…issues that physicians encounter everyday” (I-1) from repeated exposure to concepts in classroom and clinical settings, particularly while shadowing hospital ethics committee members. Others reported developing skills in clinical ethics, such as improved patient communication and utilization of resources, including clinical ethicists and spiritual services, in addressing ethically complex situations.

Regarding the role of narrative in their clinical practice, respondents explained it as cultivating a holistic sense of a patient, beyond clinical metrics—to “figure out what someone’s story is…. What is your life like? …What is this person like?” (I-10). Towards this end, individuals felt more attuned to and aware of subtle, nonverbal cues from clinical encounters, such as the cultural context of care or the emotions of patients and families. For example, one respondent made sure to incorporate “the cultural side of medicine…for the Nepali speaking patient that comes in with whatever their chief complaint is [vs.] what the true complaint is” (I-15), while another carefully observed “the mom looking down at her dress the whole time or…on her phone instead of… at her daughter who’s dying” (I-10). Multiple respondents found these approaches useful in elucidating patient values, particularly towards the end-of-life. Others also reported benefits in facilitating team communication and consensus regarding patient values for clinical decision-making. Many attributed these narrative skills to their strong background in humanities from the BEMH SC.

Lastly, respondents reported an increased ability to recognize and navigate morally and clinically complex situations in medicine without defined guidelines, or in the words of one individual (I-6) the “gray things…[that] come up in medicine where…you can’t prove it was wrong…so technically it’s ok.” For this individual, his BEMH SC experiences put him in “a position where I can step back and think about the right thing…a lot of that comes from BEMH.” As clarification, he provided an example within the realm of clinical research, when he decided against offering an innovative therapy to a patient, who likely would have consented, because of a poor risk-benefit ratio. Respondents in pathology and plastic surgery provided similar examples, where they did not settle for meeting the technical requirements of their work but actively considered patient values and potential benefits and harms to patients. One attending described this as an appreciation of the “ethical, human side of medicine in all its forms” (I-15).

#### 2. Career decisions and lifestyles

Speaking to the effect of the BEMH SC on self-care, wellness, and work-life balance, overall respondents identified physician wellness as a meaningful topic that came up in the concentration, with one individual describing it as “non-negotiable…to put your best clinical work forward, best research work forward, best kind of…self forward” (I-6). These beliefs manifested in consistent efforts to maintain sustainable work-life balance in their careers and engage in self-care and reflective practices. For some, this consisted of specific exercises (e.g., writing) developed through the BEMH SC; for others, it was related to maintaining a perspective, which allowed them to derive meaning through work by drawing attention to “the bigger picture of what we do…how it fits into a frame of larger human experience” (I-18). A few individuals felt the BEMH SC was also beneficial in identifying resources for self-care—e.g., chaplains, support groups, mental health specialists—through exposure to these services during their time in the SC.

Respondents reported that the BEMH SC influenced their choice of specialty. Multiple individuals explained it was imperative they pursued a specialty that allowed them to engage with specific humanistic themes or writing and other creative activities, which they were exposed to as medical students in the BEMH SC. Others choose specialties based on maintaining adequate work-life balance and self-care practices. Additionally, some felt that the BEMH SC supported them in their decision to pursue less competitive, often more primary care-oriented fields, such as family medicine, whereas others felt that the BEMH SC oriented them in the direction of specific fields.

Furthermore, respondents felt they had positive role models for careers in BEMH through the BEMH SC. For multiple individuals, it enabled them to realize that they could have rigorous careers in BEMH within medicine. For a select few, these experiences and relationships became the foundation for careers in or incorporating BEMH. Specific careers mentioned included physician-writers and medical news correspondents. Others integrated BEMH into their clinical and teaching responsibilities. For example, one attending created a physician wellness program for residents and another, a communication curriculum.

## Discussion

In this exploratory qualitative study—the first to examine the persisting impact of a comprehensive US BEMH SC—we observed repeated themes across responses related to (a.) student experience during medical school and (b.) post-graduate development as outlined above. Overall, they indicate that Stanford’s BEMH SC was successful in achieving the purported aims of BEMH in medical education, including foundational skills in medical professionalism and ethics, enhanced communication and observation, and self-care and reflective practices [1–9]. These perceived effects persisted beyond immediate student experience in medical school, impacting post-graduate development at various training stages. Significantly, no other BEMH curricular offering has been demonstrated to achieve similar post-graduate effects [10–12].

Additionally, our results suggest the importance of structuring BEMH in a longitudinal format with formal institutional support for scholarly projects—namely, that this format may increase the scholarly productivity of BEMH students. Since BEMH was first introduced in the US, there have been apprehensions regarding the integration of the humanities within a traditionally “hard” science and technical curriculum, especially in terms of students’ scholarly output and the development of key research skills [3, 9]. Our work may help assuage these concerns. Regarding student experience during medical school, respondents reported success in both traditional and creative scholarly venues. In their post-graduate development, some established successful careers—physician-writers, medical news correspondents, and leaders in physician wellness—related to BEMH. In particular, our results highlight the importance of funding, professional mentorship, and a requirement for scholarly productivity in promoting student and graduate success.

Furthermore, in recent years, there has been discussion about the shortcomings of BEMH coursework alone, insulated from a clinic environment, as an adequate teaching tool. For example, a 2002 qualitative analysis of syllabi for required ethics courses in US medical schools indicates a mismatch between course material and learning needs, as identified by residents, program directors, faculty, practicing physicians, and ethics committees [31, 32]. Of note, individual ethics and professionalism, referring to self-monitoring, honesty, professional demeanor, and responsibility to oneself and one’s own principles and beliefs, was a core competency markedly absent from ethics syllabi [31, 32]. Our findings indicate that the additional opportunities and structure provided by a formal BEMH SC program may better bridge the existing gap between BEMH coursework and perceived learning needs within BEMH. Regarding student experience, respondents reported both theoretical and practical exposure to BEMH through Stanford’s comprehensive BEMH SC. Speaking to later professional development, several respondents reported having an individual sense of professional integrity for morally complex situations through the BEMH SC. Such responses reflected an appreciation of the expanded role of the physician in negotiating patient values and perspectives in holistic healthcare decisions. Though not defined as such, these insights speak to the theme of professional identity formation (PIF).

PIF refers to “the foundational process one experiences during the transformation from lay person to physician—an integrative developmental process [that] involves the establishment of core values, moral principles, and self-awareness” [33] (pp246). PIF arose from conversations on professionalism, initially focusing on bioethical principles, communications skills, and behaviors, now repositioned around an integrated, reflective, and adaptive identity responsive to the dynamic and multifaceted complexities of contemporary medicine. Crucial to this process are structured reflections, often through writing and personal narratives; relationships and role modeling with regards to humanistic and self-care; and a community of peers and colleagues to promote these practices [34, 35]. Indeed, recent perspectives from leaders in medical humanities education promote that the humanities have a unique place in enabling trainees to better recognize and constructively approach the limitations, complexities, and thereby the uncertainty and discomforts of contemporary medicine [36, 37]. Medical professionalism—defined by traits such as empathy and altruism,—has long been a desired outcome for BEMH. Our findings indicate that the opportunities and structure for reflection, mentorship, and community provided by a BEMH SC may promote new notions of professionalism, centered around identify formation, which better address previously identified core competencies [32].

### Limitations

Our study has several limitations. First, our findings only represent respondents’ perception of the impacts of the BEMH SC. However, as such impacts are often intangible, difficult to measure, or previously unreported, they are best captured—initially—in an exploratory qualitative study. All reported findings were discussed by multiple respondents. However, for some individuals, there was minimal impact in a particular domain. Others had difficulty discerning which impacts were attributable to the BEMH SC vs. pre-existing qualities or other aspects of training. At minimum, the BEMH SC provided a comprehensive, structured opportunity that otherwise would not have existed for students to develop interests and skills in BEMH. Second, the generalizability of our findings is limited, due to a possible selection bias among participants who choose the BEMH SC and subsequently elected to participate in this study. Such selection bias may have resulted in a positive skew in perceived impacts. Additionally, this is a study specific to graduates of a single institution. However, as the first study to examine the perceived impacts of a BEMH SC, our study was designed to identify themes that could be subsequently referred to in future studies. BEMH SCs are still an emerging trend with relatively few programs in the US, most with unique characteristics. Future research could come in the form of multi-institutional studies, comparing and contrasting and identifying commonalities across programs. Lastly, BEMH itself is not a coherent, fixed discipline, but rather a highly diverse field with a contested definition, which could complicate cross-study comparisons [2–5, 7–9].

## Conclusion

A comprehensive US BEMH SC achieved the purported aims of BEMH in medical education. Medical graduates perceived these effects to persist into clinical practice. Similar post-graduate effects have not been demonstrated for other BEMH curricular offerings. The structure and format of a SC may offer additional advantages in promoting student scholarly skill and productivity, career development, and professional identity formation—core competencies identified across clinical training and ethics programs. Though time and cost considerations may be prohibitive in some cases, medical educators and administrators seeking to fully reap the benefits of BEMH in medical education should consider a comprehensive BEMH SC as a particularly promising venue, achieving a range of desired immediate and post-graduate effects.

## Additional files

Additional file 1: Telephone Survey. This structured telephone survey was conducted prior to each interview and consists of questions related to participant characteristics (e.g., entering and graduating years, choice of specialty) as well as validated demographic questions from the US census. (DOCX 109 kb)

Additional file 2: Interview Guide. The semi-structured interview guide probed relevant perceived impacts for biomedical ethics and medical humanities as well as Scholarly Concentration programs derived from the literature. Interviews were left intentionally semi-structured to facilitate an open-ended examination of participants’ experiences and perspectives beyond identified outcomes. However, this guide provides a basic framework for interviews conducted in the course of this study. (DOCX 87 kb)

## Abbreviations

BEMH: Bioethics and Medical Humanities
IRR: Inter-rater Reliability
SC: Scholarly Concentration
US: United States
