# Supplementary material for: The long-term impact of a comprehensive scholarly concentration program in biomedical ethics and medical humanities
Source: BMC Med Educ. 2018 Aug 28;18:204. doi: 10.1186/s12909-018-1311-2 (PMC6114241; doi:10.1186/s12909-018-1311-2)
Supplement: Supplementary file 1 — Telephone Survey. This structured telephone survey was conducted prior to each interview and consists of questions related to participant characteristics (e.g., entering and graduating years, choice of specialty) as well as validated demographic questions from the US census. (DOCX 109 kb) [file 12909_2018_1311_MOESM1_ESM.docx]

Participant Survey

1. When did you first enter the MD program (YYYY)?

2. When did you graduate from the MD program (YYYY)?

3. Do you have any additional graduate degrees besides your MD? Was it/were they earned jointly with your MD?

|  | Check all that apply | |
| --- | --- | --- |
|  | Joint with MD | Separately from MD |
| a. Professional doctorate (e.g., JD)? |  |  |
| b. Professional master’s degree (e.g., MBA, MPH)? |  |  |
| c. Research degree (PhD, PsyD, MS, MSc, etc.)? |  |  |

4. Did you do a Scholarly Concentration in BEMH?

5. Did you complete a Medical Scholars project in the BEMH Scholarly Concentration?

6. Before declaring your Scholarly Concentration in BEMH/completing your MedScholars project in BEMH did you have any experiences (undergraduate or advanced degrees, coursework, research experience) in…?

|  | Check all that apply |
| --- | --- |
|  |  |
| a. Biomedical Ethics and Medical Humanities |  |
| b. A related field (History of Science, etc.), specify: |  |
| _______________________________________________ |  |

7. On a scale of 1-5, with 1 being not involved beyond requirements and 5 being very involvement, what was your overall research involvement as a medical student?

| Not involved beyond requirement | Minimally  involved | Somewhat involved | Involved | Very involved |
| --- | --- | --- | --- | --- |
| 1 | 2 | 3 | 4 | 5 |

8. On a scale of 1-5, with 1 being not involved beyond requirements and 5 being very involvement, what was your involvement in BEMH activities as a medical students?

| Not involved | Minimally involved | Somewhat involved | Involved | Very involved |
| --- | --- | --- | --- | --- |
| 1 | 2 | 3 | 4 | 5 |

9. What is your current professional title (e.g., resident, attending, etc.)?

10. What is your current specialty?

11. Do your current job responsibilities include…?

|  | Check all that apply |
| --- | --- |
|  |  |
| a. Patient care |  |
| b. Research and/or other kinds of scholarship |  |
| c. Teaching |  |
| d. Health management and administration |  |
| e. Public health and policy |  |
| f. Other, specify |  |
| ___________________________________________________ |  |

12. Of the following four, which best describe your primary place of employment? You can pick as many as apply.

|  | Check all that apply |
| --- | --- |
|  |  |
| a. Academic medical center |  |
| b. For-profit hospital |  |
| c. Non-profit hospital |  |
| f. Other, specify: |  |
| ___________________________________________________ |  |

**Demographics**

1. What is your sex?

Male

Female

2. What is your age?

3. Are you of Hispanic, Latino, or Spanish origin?

Yes

No

4. Were you born in the United States, on the island of Puerto Rico, or in another country?

United States

Puerto Rico

Another country

4. Of the following six, which best describe your race? You can pick as many as apply.

White (European, Irish, Italian, Arab, Middle Eastern, etc.)

Black or African-American (Kenyan, Nigerian, Haitian, etc.)

Asian or Asian-American (Asian Indian, Chinese, Filipino, Vietnamese, etc.)

Native American/American Indian/Alaska Native

Pacific Islander/Native Hawaiian

Other, specify:___________________________________________________

**Thank you for your time!**
