# Supplementary material for: The long-term impact of a comprehensive scholarly concentration program in biomedical ethics and medical humanities
Source: BMC Med Educ. 2018 Aug 28;18:204. doi: 10.1186/s12909-018-1311-2 (PMC6114241; doi:10.1186/s12909-018-1311-2)
Supplement: Supplementary file 2 — Interview Guide. The semi-structured interview guide probed relevant perceived impacts for biomedical ethics and medical humanities as well as Scholarly Concentration programs derived from the literature. Interviews were left intentionally semi-structured to facilitate an open-ended examination of participants’ experiences and perspectives beyond identified outcomes. However, this guide provides a basic framework for interviews conducted in the course of this study. (DOCX 87 kb) [file 12909_2018_1311_MOESM2_ESM.docx]

Thank you very much for agreeing to speak with me today. Before we start, I just wanted to quickly introduce myself – my name is Emily Liu, and I’m a Stanford medical student concentrating in the Biomedical Ethics and Medical Humanities (BEMH) Scholarly Concentration.

I’d like to begin by obtaining your oral consent to the study. You should also have received a copy of this information by email already. Please listen carefully.

[Oral Consent Script]

Do you have any questions at this point?

Do you consent to participate in this study?

Do you consent to being audio-recorded?

Alright, then I’ll begin recording now.

I’d like to start by asking a few basic background questions before proceeding to the interview.

[Survey questions]

Wonderful, now let’s proceed to the interview.

Basics

What stood out for you about the BEMH Scholarly Concentration/BEMH at Stanford?

Goal: as written

- *What did you think about the BEMH curriculum/classes?*
  - *Goal: pros, cons, challenges*
- *What kinds of experiences did you have in research (e.g., description of MedScholars project) through BEMH?*
  - *Goals: pros, cons, challenges*
- *Tell me about your extracurricular activities in the BEMH Scholarly Concentration/ BEMH at Stanford.*
  - *Goals: pros, cons, and challenges*
- *Tell me about the mentorship and advising you received through the Scholarly Concentration/BEMH at Stanford.*
- *How did the Scholarly Concentration/experiences in BEMH at Stanford contribute to your personal happiness and well-being as a medical student, if at all?*
- *In hindsight, how could your experiences in the Scholarly Concentration/with BEMH at Stanford have been better?*
  - *Goals: action items, concern: listing question?*

Professional Development

Can you describe your path since medical school?

Goal: basic description

How have your experiences in the BEMH Scholarly Concentration/BEMH at Stanford influenced your career trajectory?

Goal: determine areas of greatest impact re: career trajectory, etc.

- *How did your time in the concentration/with BEMH at Stanford influence your career choices (as a physician, researcher, policy maker, advocate, administrator, writer, artist, etc.), if at all?*
- *How did mentoring and advising through the concentration/BEMH at Stanford impact your professional advancement, if at all?*
- *How do you apply the knowledge, experience, and skills you gained as a medical student in the concentration/through BEMH at Stanford now, if at all (as a physician, researcher, policy maker, advocate, administrator, writer, artist, etc.)?*
  - *Goal: determine areas of greatest impact re: professional skill development*
- *How did your time in the concentration/with BEMH at Stanford influence your views regarding work-life balance?*

Clinical practice

How have your previous experiences in the BEMH Scholarly Concentration/with BEMH at Stanford impacted your clinical practice?

- *How have they impacted your ability to empathize with patients?*
  - *Goal: impact of BEMH on physician empathy*
- *How have they impacted your ability to communicate with patients?*
  - *Goal: impact of BEMH on physician-patient communication*
- *How have they contributed to your clinical observational skills?*
  - *Goal: impact of BEMH on clinical observational skills*
- *How have they contributed to your teamwork abilities?*
  - *Goal: impact of BEMH on teamwork skills*
- *How did they prepare you to handle ethical issues that emerge in clinical research and practice?*
  - *Goal: impact of BEMH on professional ethics*
- *Have they impacted your clinical practice in other ways?*
  - *Goal: other notable impacts of BEMH re: clinical practice*
- *Can you give me an example or perhaps share an interesting experience with me?*
  - *Goal: illustrative example for paper, etc.*

Great, now I have few demographic questions I’d like to ask for statistical purposes.

[Demographic questions]

Closing

Thank you for opening up to me today. I’ve really enjoyed learning more about your experiences as a medical student and your professional trajectory since then. Before I let you go, is there anything else that you would like to share with me, either that I have or haven’t asked about? Do you have any questions for me?

Great, well just to reiterate, your comments will be anonymized in the transcription process. If you have any follow up questions regarding what we have discussed, please do not hesitate to contact me. Thank you so much for your time!
